# Integrative bulk and single-cell transcriptomic analysis identify an ac4C-related signature in lung adenocarcinoma

**DOI:** 10.7150/jca.135531

**Published:** 2026-07-13

**Authors:** Shuo Wang, Changqing Yang, Xingkai Wang, Zhigang Zhu, Yuxin Xie, Rui Wang, Dan Liu, Jing Feng

**Affiliations:** 1 Department of Respiratory and Critical Care Medicine, Tianjin Medical University General Hospital, Tianjin, 300052, China.; 2 Tianjin Institute of Urology, The Second Hospital of Tianjin Medical University, Tianjin, China.; 3 Department of Respiratory and Critical Care Medicine, Bishan Hospital of Chongqing Medical University, Chongqing 402760, China.

**Keywords:** lung adenocarcinoma, single-cell analysis, ac4C, PLK1, prognostic signature

## Abstract

**Background:**

N4-acetylcytidine (ac4C) RNA modification is a critical epitranscriptomic regulator of cancer progression, yet its specific biological functions and regulatory patterns in lung adenocarcinoma (LUAD) remain poorly understood. This study aims to characterize the clinical relevance and potential regulatory patterns of ac4C-related features in the LUAD tumor microenvironment (TME).

**Methods:**

We integrated bulk RNA-seq data from TCGA and single-cell RNA-seq (scRNA-seq) data from GEO. Weighted gene co-expression network analysis (WGCNA) identified prognosis-related modules. A robust six-gene ac4C-related signature was derived using three consensus machine learning algorithms: LASSO, Random Forest, and SVM-RFE. The signature was further validated using pseudotime trajectory inference, CellChat-based intercellular communication analysis, and *in vitro* functional assays.

**Results:**

The six-gene signature effectively stratified LUAD patients, identifying a high-risk subgroup characterized by poor survival and frequent TP53 mutations. Single-cell analysis suggested that high ac4C-related signature scores were associated with advanced malignant states and enhanced predicted pro-tumorigenic signaling within the TME, particularly involving the EGF, TGFB, and MIF pathways. Pharmacogenomic modeling identified increased sensitivity to CDK and PLK1 inhibitors in high-risk patients. Experimentally, PLK1 silencing significantly suppressed LUAD cell proliferation and migration.

**Conclusions:**

This study identifies a potential ac4C-related prognostic signature associated with malignant cell states and predicted communication patterns within the TME, and suggests PLK1 as a candidate therapeutic target in LUAD.

## Introduction

Lung cancer continues to be the leading cause of cancer mortality worldwide. Among various histological types, lung adenocarcinoma (LUAD) is the most common subtype^1^, ^2^. Despite major progress in targeted therapies and immune-based treatments, survival outcomes for LUAD patients are still limited^3^, ^4^. A major obstacle to effective treatment is the profound inter- and intra-tumoral heterogeneity, which drives heterogeneous disease trajectories and contributes to therapeutic resistance^5^, ^6^. Accordingly, robust biomarkers are needed to enable precise risk stratification and guide personalized clinical management.

Recent advances in epitranscriptomics have revealed that post-transcriptional RNA modifications significantly influence gene expression and participate in numerous pathological processes, including carcinogenesis^7^, ^8^. Among over 170 known RNA modifications, N4-acetylcytidine (ac4C) has emerged as an evolutionarily conserved mark that regulates mRNA stability and translation efficiency^9^. Catalyzed by its only known "writer", N-acetyltransferase 10 (NAT10)^10^, ac4C modification is increasingly linked to malignant behaviors such as cell proliferation, metastasis, and metabolic reprogramming in various cancers^11^, ^12^. Given accumulating evidence that ac4C modification is critically involved in tumorigenesis^13^-^15^, this epitranscriptomic mark has emerged as a promising target for cancer diagnosis and therapy. However, the functional significance of ac4C modification in LUAD and its regulatory networks, particularly in relation to the tumor microenvironment and cellular heterogeneity, remain poorly defined.

While bulk RNA-sequencing (RNA-seq) provides valuable insights into general molecular profiles, it often obscures the complex heterogeneity within the malignant compartment. By contrast, single-cell RNA sequencing enables high resolution profiling of the tumor microenvironment, clarifying the contributions of distinct cell subsets and their communication networks^16^. Integrating bulk and single-cell transcriptomic data can thus provide a comprehensive view of the genomic and cellular landscape of LUAD^17^, ^18^.

In this study, we integrated multi-omics datasets to define the clinical and biological significance of ac4C modification in LUAD. By employing complementary machine learning algorithms, we developed an ac4C-related prognostic signature and utilized scRNA-seq data to link this signature to malignant cell states and TME remodeling. Furthermore, we assessed candidate therapeutic vulnerabilities to identify potential drugs for high-risk patients. Our data expand current understanding of ac4C-related regulation in LUAD and suggest an ac4C-based biomarker with potential utility for risk prediction and personalized therapeutic decision making.

## Materials and Methods

### Data Acquisition and Pre-processing

Bulk RNA-seq data, matched clinical annotations, and somatic mutation profiles for the TCGA LUAD cohort were retrieved using the TCGAbiolinks R package (v2.34.1)^19^. After matching RNA-seq profiles with survival annotations and excluding records without complete overall survival information, 645 TCGA-LUAD RNA-seq samples were retained for downstream analyses. Adjacent normal tissues were used only for tumor-normal differential expression analysis. For multivariate Cox regression, 485 patients with complete clinicopathological information were included. The independent GSE13213 cohort was used for external validation, and GSE131907 was used for single-cell analysis^20^, ^21^. Bulk RNA-seq expression matrices were normalized to transcripts per million (TPM). The initial ac4C-related gene set (acRG) was curated from published ac4C-RIP-seq data by collecting experimentally identified ac4C-enriched transcripts^9^. This acRG set was used as a reference to explore ac4C-associated transcriptional programs in LUAD.

### Weighted Gene Co-expression Network Analysis (WGCNA)

The WGCNA R package was applied to the TCGA-LUAD expression matrix of acRGs to infer co-expression modules^22^. Candidate soft-thresholding powers from 1 to 10 and from 12 to 20 with an interval of 2 were evaluated using the scale-free topology criterion, and the soft-thresholding power was set to 6. Co-expression modules were constructed using an unsigned topological overlap matrix. Modules were detected using the dynamic tree cut algorithm with a minimum module size of 30, a reassign threshold of 0, and a merge cut height of 0.25. To evaluate clinical significance, each module eigengene was entered into a univariate Cox proportional hazards model, and modules with P < 0.05 were considered significantly associated with overall survival.

### Identification of the ac4C-Related Signature via Machine Learning

Differentially expressed genes (DEGs) were extracted from TCGA-LUAD cohort, representing LUAD tumor samples versus adjacent normal tissues, using limma package (v3.54.2) (adjusted P < 0.05, |log2 fold change| > 1)^23^. Candidate genes were extracted by intersecting the genes from previously identified risk-associated WGCNA modules (MEblue, MEgreen) with upregulated DEGs. To obtain a robust feature set, candidate genes were further prioritized using three complementary machine learning approaches^24^-^26^. LASSO regression was performed using the glmnet package, with the optimal penalty parameter selected by 10-fold cross-validation. Random Forest was implemented using the randomForest package, and SVM recursive feature elimination was conducted using the e1071 package^27^-^30^. Finally, six consensus genes were selected from the intersection of features identified by all three algorithms to construct the ac4C-related signature. The risk score was calculated as Σ Coefᵢ × Expᵢ, using coefficients derived from the TCGA-LUAD cohort and normalized gene expression values. Patients were stratified into high- and low-risk groups by the median risk score within each cohort.

The robustness of the six-gene signature was assessed by bootstrap internal validation in the TCGA-LUAD cohort and external validation in the independent GSE13213 cohort using the coefficients derived from the TCGA-LUAD model. Prognostic performance and independence were evaluated by Kaplan-Meier analysis, Cox regression, Harrell's C-index, and time-dependent ROC analysis. Age, sex, pathological stage, and the standardized risk score were included in the multivariate Cox model.

### Functional and Genomic Annotation of the Signature

Hallmark pathway enrichment was performed using clusterProfiler (v4.6.2), with Benjamini-Hochberg-adjusted P values used where applicable^31^. Somatic mutation profiles were analyzed using maftools (v2.20.0), and mutation frequency differences between high- and low-risk subgroups were assessed by Fisher's exact test and reported as exploratory nominal P values^32^.

### Processing and Analysis of Single-cell Data

Single-cell data were processed according to the standard Seurat workflow in R (v4.4.0)^33^. Cells with nFeature_RNA > 200, nCount_RNA > 500, and mitochondrial gene percentage < 25% were retained for downstream analysis. The amount of ambient RNA contamination was calculated using the decontX package, and cells with contamination score ≥ 0.2 were filtered away^34^. Batch effects were corrected with Harmony (v1.2.0), and putative doublets were identified and excluded using DoubletFinder (v2.0.3)^35^, ^36^. During the gene expression normalization process, the SCTransform command was used^37^. We classified cell types using well accepted marker gene panels, with annotation guided by published references. Malignant epithelial cells were inferred using inferCNV (v1.14.2), with non-epithelial immune and stromal cells used as reference populations. Epithelial clusters showing broad chromosome-level CNV alterations together with epithelial marker expression were classified as malignant^38^.

### Pseudotime Trajectory and Cell Communication Analysis

The malignant cell population was subsetted for further downstream analysis. Cell-level ac4C-related signature scores were derived from the single-cell expression matrix using Seurat's AddModuleScore. Malignant cells were stratified into signature-high and signature-low groups according to the median ac4C-related signature score. Pseudotime ordering of malignant cell subclusters was conducted using Monocle to model their developmental dynamics^39^. The root state was assigned to the branch with relatively low ac4C-related signature scores and lower proliferative activity. Intercellular communication was inferred using CellChat (v1.6.1) with default settings and the CellChatDB.human ligand receptor database^40^.

### Drug Sensitivity Prediction

Drug sensitivity analysis was implemented using the GDSC public database^41^. In order to impute the sensitivity values (based on IC50 values) for each patient sample, oncoPredict R package (v1.2) was used^28^. Differences between groups were tested by t test. Given the exploratory nature of the *in silico* drug sensitivity screen, nominal P < 0.05 was used, and the results were interpreted as hypothesis-generating.

### Cell Culture and siRNA Transfection

A549 and H1299 LUAD cells were maintained in RPMI 1640 (KeyGEN BioTECH, KGL1503-500) containing 10% fetal bovine serum (Corning) and 1% penicillin streptomycin (Gibco) under standard conditions (37 °C, 5% CO₂). For siRNA-mediated knockdown, cells were seeded in 6-well plates (3 × 10⁵ cells/well) and transfected at ~50-70% confluence with a non-targeting control siRNA (siNC) or two independent PLK1-targeting siRNAs (si-PLK1 #1 and si-PLK1 #2) using Lipofectamine RNAiMAX (Invitrogen, 13778150) in Opti-MEM (Gibco, 31985062) according to the manufacturer's instructions. siRNA and Lipofectamine RNAiMAX were prepared separately in Opti-MEM, then combined and allowed to stand for 10 to 20 min at room temperature to form transfection complexes. The mixture was added to cells to yield a final siRNA concentration of 50 nM. The siRNA duplexes (5'→3') were: si-PLK1 #1, sense GCACAUACCGCCUGAGUCU and antisense AGACUCAGGCGGUAUGUGC. si-PLK1 #2, sense CCACCAAGGUUUUCGAUUG and antisense CAAUCGAAAACCUUGGUGG^42^. Complete medium was restored 6 h post transfection.

### RT-qPCR and *In Vitro* Functional Assays

Total RNA was isolated at 24 h post siRNA transfection with an RNA purification kit (Esunbio, RN001). After quantification, cDNA was synthesized using HiScript III RT SuperMix for qPCR with gDNA removal (Vazyme, R323-01). Quantitative PCR was carried out using ChamQ Universal SYBR qPCR Master Mix (Vazyme, Q711-02) and gene specific primers. Transcript levels were normalized to GAPDH and calculated with the 2^⁻ΔΔCt^ method^43^.

Cell growth was quantified by the CCK-8 assay. After transfection, A549 cells (1.5 × 10^3^ per well) and H1299 cells (2 × 10^3^ per well) were plated in 96-well plates, and CCK-8 reagent (Beyotime, C0038) was applied at 0, 24, 48, 72, and 96 h. Absorbance was recorded at 450 nm. Colony formation assays were conducted by seeding transfected cells at low density and culturing for 10-14 days before fixation, staining, and colony counting. Wound-healing assays were performed by generating a scratch in near-confluent monolayers, followed by serial imaging and quantification of wound closure.

### Statistical Analysis

All statistical analyses were conducted in R (v4.4.3), and two-sided *P* values below 0.05 were considered statistically significant unless stated otherwise. For each analysis, the corresponding statistical test is described in the relevant Methods sections and figure legends.

## Results

### Discovery of Survival-Associated Gene Modules in LUAD

**Figure 1:**
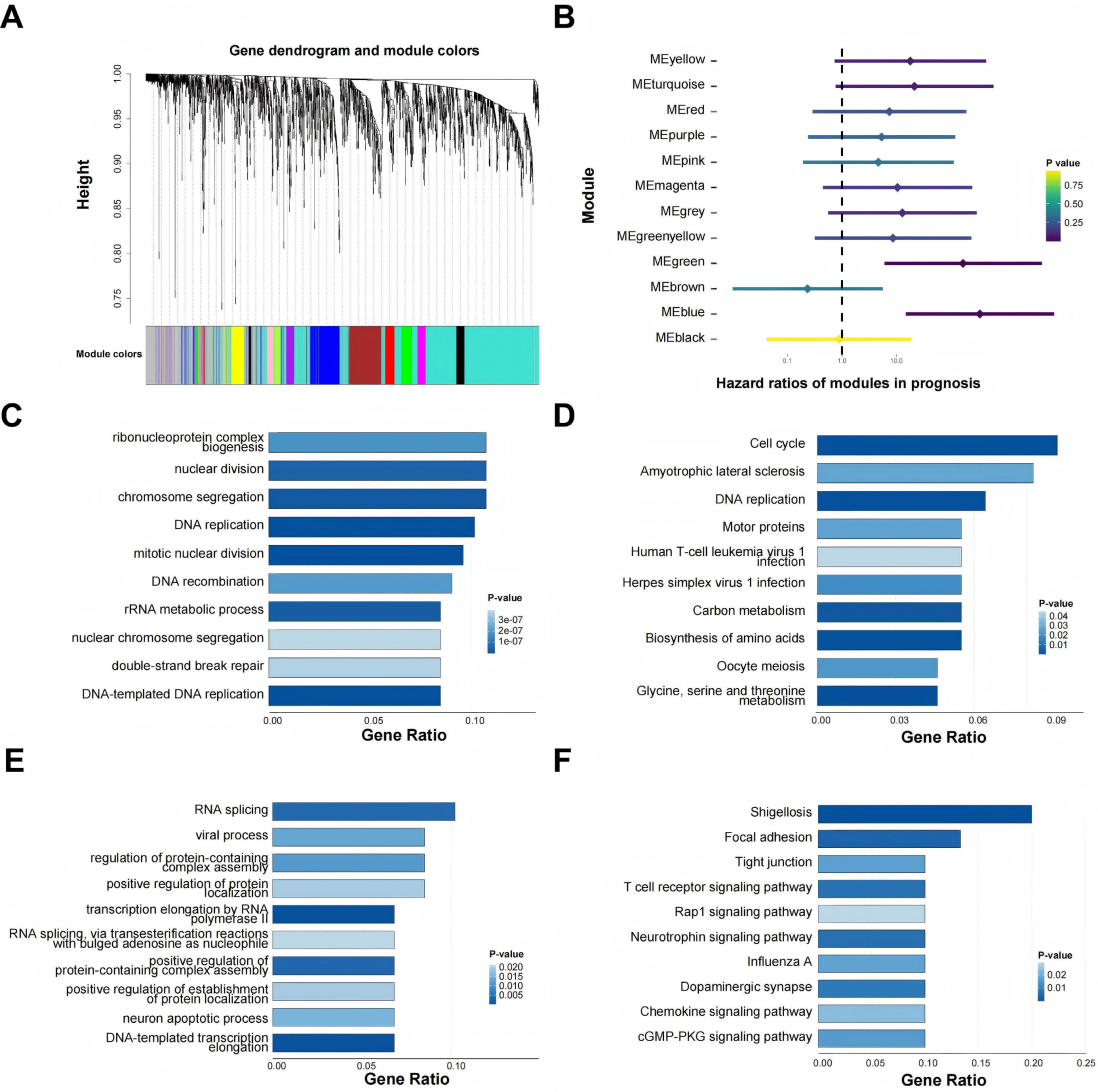
** Identification and functional annotation of prognosis-related gene modules in LUAD. (A)** Gene dendrogram and module colors identified by WGCNA. Dendrograms show the relationship between all genes and modules; the colored bar below shows the assignment of genes to co-expression modules. **(B)** Forest plot for displaying the Hazard Ratios (HRs) of the identified gene modules in prognosis. MEblue and MEgreen modules were identified as significant risk factors in LUAD (HR > 1). The color of the point estimate represents the P value. **(C, D)** Gene Ontology (GO) and Kyoto Encyclopedia of Genes and Genomes (KEGG) pathway enrichment analyses for the risk-associated MEblue module. **(E, F)** Gene Ontology (GO) and Kyoto Encyclopedia of Genes and Genomes (KEGG) pathway enrichment analyses for the risk-associated MEgreen module.

Integrating bulk and single-cell transcriptomes enables a more holistic characterization of LUAD at both genomic and cellular levels^44^. Using the expression profiles of acRGs in the TCGA-LUAD cohort, multiple distinct gene modules were identified and visualized (Figure 1A). When module eigengenes were tested in univariate Cox models, MEblue and MEgreen each showed a significant association with inferior survival (HR > 1, P < 0.05), leading us to focus on these two modules as risk related candidates (Figure 1B). Functional interpretation revealed distinct biological themes. Genes within MEblue mapped mainly to proliferation linked pathways, with prominent enrichment for cell cycle control and DNA replication (Figures 1C-D). MEgreen, however, was characterized by enrichment in RNA splicing and cell adhesion programs together with immune associated signaling (Figures 1E-F). Taken together, the data suggest that heightened proliferative activity and perturbed RNA processing may jointly contribute to the unfavorable clinical course observed in LUAD.

### Development of a Robust ac4C-Related Gene Signature

**Figure 2:**
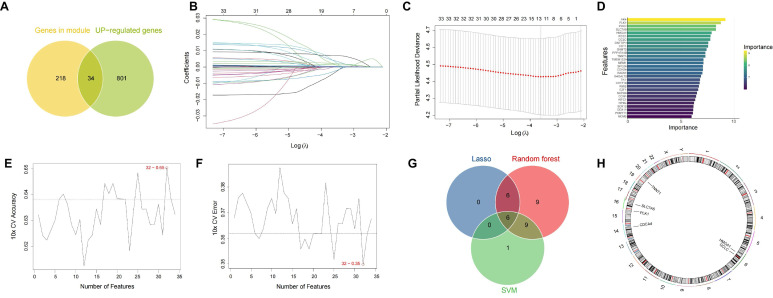
** Identification of the ac4C-related gene signature via three machine learning algorithms. (A)** Venn diagram based on the intersection of blue module and green module genes and upregulated differentially expressed genes (DEGs). **(B-C)** Feature selection using the LASSO regression model. **(B)** LASSO coefficient profiles of candidate genes. **(C)** A plot depicting the relationship between partial likelihood deviance and log(λ) determined by 10-fold cross-validation. **(D)** Feature importance ranking of candidate genes as determined by the Random Forest algorithm. **(E-F)** Feature selection using the SVM-RFE algorithm. **(E)** A plot depicting the relationship between the number of selected features and 10-fold cross-validation accuracy. **(F)** A plot depicting the relationship between the number of features and 10-fold cross-validation error. **(G)** Venn diagram showing the intersection of features selected by the LASSO, Random Forest, and SVM-RFE algorithms. **(H)** Circos plot depicting the chromosomal locations of the six signature genes.

We next obtained 34 candidate prognostic genes by intersecting the genes from risk-associated MEblue and MEgreen modules and upregulated DEGs between LUAD tumors and adjacent normal lung tissues from TCGA-LUAD cohort (Figure 2A). Then we used three machine learning algorithms to screen these candidates based on their respective advantages: LASSO regression (Figure 2B, 2C), Random Forest (Figure 2D) and SVM-RFE (Figure 2E, 2F). Finally, by integrating the results of the three algorithms, we identified six consensus genes, CDCA4, GCLC, HMGA1, PLK1, SLC7A5, and TNNT1, as the final ac4C-related signature (Figure 2G). Their chromosomal locations and reported biological or cancer-related roles are summarized in Figure 2H and Supplementary Table S3. This six-gene signature was then used for subsequent risk stratification. Additional validation supported the prognostic utility of the signature. Bootstrap internal validation yielded an optimism-corrected C-index of 0.629, and the signature stratified overall survival in the independent GSE13213 cohort (P = 0.0026; Supplementary Figure S2A-D; Table S1). In the TCGA-LUAD cohort, the standardized risk score remained independently associated with poorer overall survival after adjustment for age, sex, and pathological stage (HR = 1.558, 95% CI: 1.343-1.806, P = 4.60 × 10⁻⁹; Supplementary Figure S2E-F; Table S2). NAT10 expression was positively correlated with the risk score (Spearman's r = 0.301, P = 5.49 × 10⁻¹²; Supplementary Figure S2G).

### Functional and Genomic Characterization of the ac4C Risk Signature

**Figure 3:**
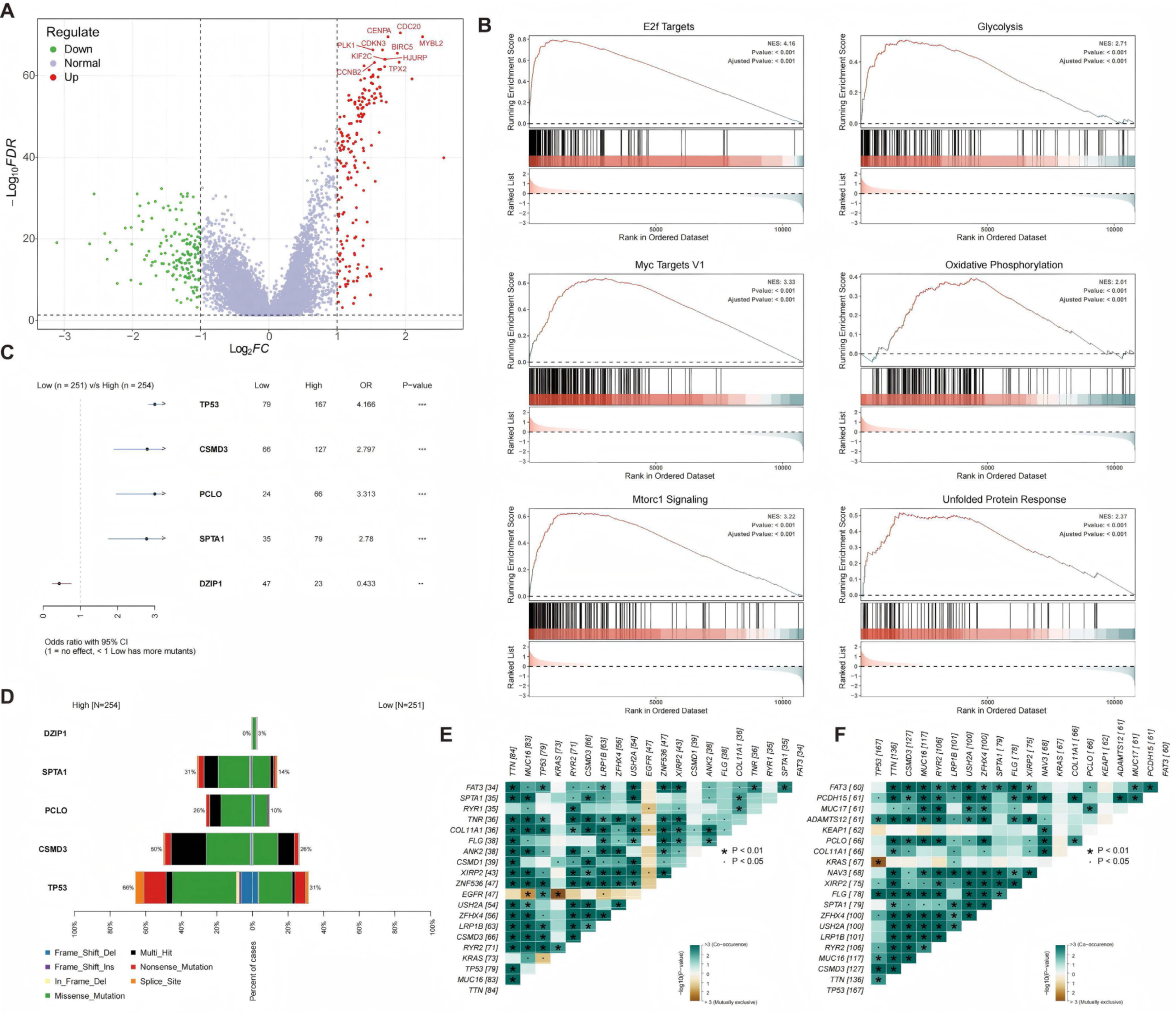
** Functional and genomic characterization of the ac4C signature. (A)** Volcano plot showing differentially expressed genes (DEGs) between the high- and low-risk groups. Red dots and green dots represent upregulated genes and downregulated genes, respectively. **(B)** Gene Set Enrichment Analysis (GSEA) plots of significantly enriched hallmark pathways, including 'E2F Targets', 'Glycolysis', 'MYC Targets V1', 'Oxidative Phosphorylation', 'mTORC1 Signaling', and 'Unfolded Protein Response', in the high-risk group.** (C)** Forest plot showing differentially mutated genes between the high- and low-risk groups (odds ratio, OR). **(D)** Waterfall plot depicting the somatic mutation landscape of the high- and low-risk groups.** (E, F)** Landscape of gene co-mutations in the low-risk group **(E)** and the high-risk group **(F)**. Green squares and brown squares represent significant co-occurrence and mutual exclusivity, respectively. The asterisks represent the statistical significance level.

We next characterized the molecular landscapes of the identified patient subgroups. Differential expression analysis was performed (Figure 3A). GSEA demonstrated that the high-risk group was significantly enriched in oncogenic pathways, including 'E2F Targets', 'MYC Targets', and 'mTORC1 Signaling', alongside activated metabolic programs such as glycolysis (Figure 3B). We compared somatic mutation patterns between the high- and low-risk subgroups. Fisher's exact test showed higher mutation frequencies of TP53, CSMD3, PCLO, and SPTA1 in the high-risk group, whereas DZIP1 was enriched in the low-risk group (Figure 3C). The overall mutation landscapes are summarized in Figure 3D. Co-occurring alterations were more frequent in the high-risk subgroup than in the low-risk subgroup (Figures 3E-F), indicating a distinct mutational context that provides a genomic basis for the aggressive behavior and poor prognosis of high-risk tumors.

### Single-cell Profiling Delineates the LUAD Tumor Microenvironment

**Figure 4:**
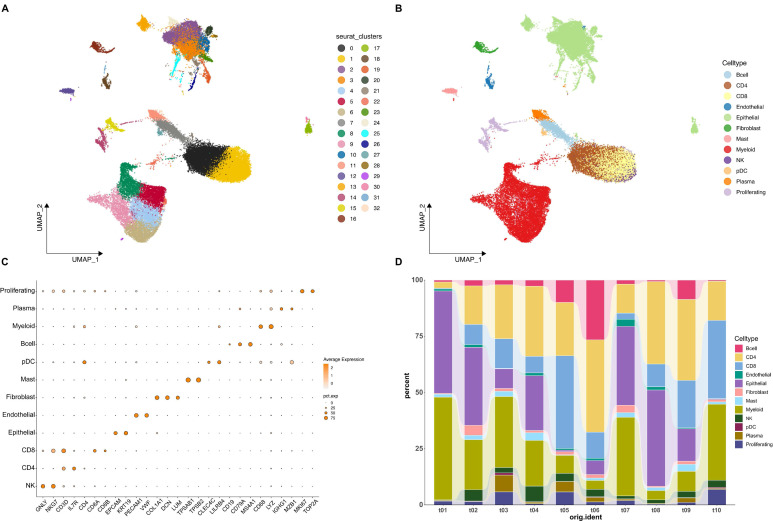
** Single-cell transcriptomic landscape of LUAD. (A)** Uniform Manifold Approximation and Projection (UMAP) plot of all cells from tumor samples, colored by unsupervised Seurat clusters. **(B)** UMAP plot of all cells, colored by the 12 annotated cell types.** (C)** Dot plot showing the expression levels of canonical marker genes used to identify the different cell populations. Dot size represents the percentage of cells in a cluster expressing the gene, and color intensity represents the average expression level. **(D)** Stacked bar chart illustrating the proportion of each cell type across the different patient samples included in the analysis.

To elucidate the cellular basis of our bulk transcriptomic findings, we analyzed LUAD samples at single-cell resolution. After quality control and data integration, 53,859 cells from 10 patient samples were retained for further analysis. After UMAP embedding, cells segregated into 33 clusters (Figure 4A). The malignant status of the epithelial cell clusters was subsequently confirmed by inferring large-scale copy number variations (CNVs), which distinguished them from normal epithelial and other non-malignant cell types (Supplementary Figure S1). We next assigned biological identities to the 33 clusters and grouped them into 12 major cell types. These included CD4+ and CD8+ T cells, B cells, NK cells, myeloid cells, plasma cells, epithelial cells, endothelial cells, fibroblasts, mast cells, plasmacytoid dendritic cells, and a distinct proliferating cell population (Figure 4B). Cell type labels were determined using established marker gene expression patterns (Figure 4C). For example, the expression of CD3D and IL7R identified T cells; the expression of EPCAM and KRT19 identified epithelial cells; the expression of COL1A1 and DCN identified fibroblasts; and the expression of MKI67 and TOP2A identified proliferating cells (Figure 4C). Subsequently, we investigated the cellular composition of all samples, and results showed that the proportion of different cell populations varied markedly among different patient samples (Figure 4D).

### Pseudotime Analysis Links the ac4C-Related Signature to Malignant Cell States

**Figure 5:**
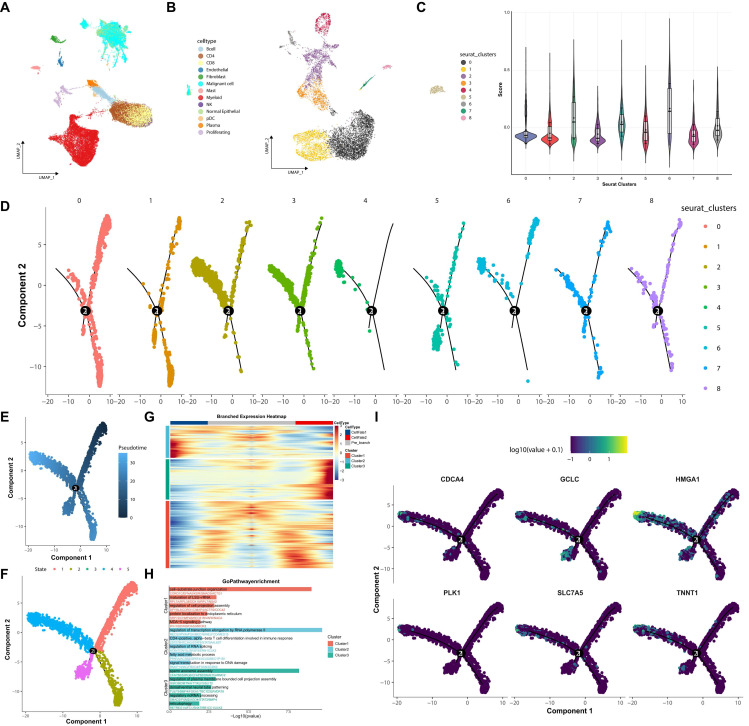
** Pseudotime trajectory analysis reveals developmental dynamics of malignant cells. (A)** UMAP plot of malignant cells. **(B)** UMAP plot of subclustering of malignant cell population into nine subgroups. **(C)** Violin plot of ac4C-related signature scores across malignant subclusters. **(D)** Inferred pseudotime trajectory overlaid on individual malignant subclusters. **(E)** Trajectory colored by pseudotime progression.** (F)** Trajectory colored by distinct cell states. **(G)** Branched expression heatmap of genes at a trajectory branch point. **(H)** Gene Ontology (GO) enrichment analysis for the three gene clusters displayed in **(G)**. **(I)** Expression of the ac4C-related signature genes visualized along the developmental trajectory.

To dissect intratumoral heterogeneity within the malignant compartment, we first delineated malignant cells from the overall tumor microenvironment, and then these cells were sub-clustered into nine subgroups (Figure 5A-B). Per-cell ac4C-related signature scores were generated from the signature, revealing significant enrichment in clusters 2, 4, and 6 (Figure 5C). Pseudotime trajectory analysis further inferred a branched developmental structure among malignant cells (Figure 5D-F). These signature-high clusters were predominantly distributed toward the terminal region of a specific inferred trajectory, suggesting a potential association with more advanced malignant cell states. Branch-dependent differential expression analysis identified transcriptional programs associated with this trajectory (Figure 5G). Gene Ontology enrichment of the branch-associated module implicated processes including ribosome biogenesis and positive regulation of cell migration (Figure 5H). Consistent with this, signature genes like PLK1 and CDCA4 were progressively upregulated along this trajectory (Figure 5I).

### Signature-High Malignant Cells Show Enhanced Predicted Pro-Tumorigenic Signaling

**Figure 6:**
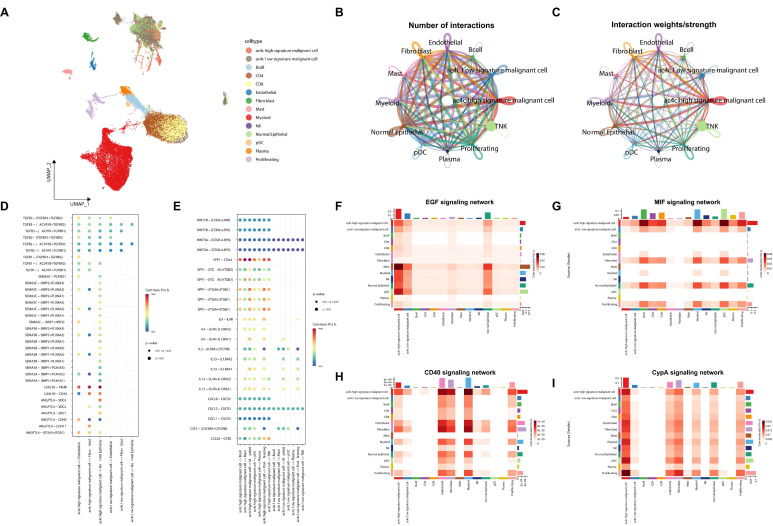
** Signature-high malignant cells show enhanced predicted pro-tumorigenic signaling. (A)** UMAP plot showing division of malignant cells into signature-high and signature-low groups within the tumor microenvironment. **(B, C)** Global CellChat network visualized as a UMAP plot showing the overall number **(B)** and strength **(C)** of interactions within the TME.** (D, E)** Bubble plots of key ligand-receptor interactions, highlighting increased TGFB signaling in the signature-high group.** (F-I)** Heatmaps showing comparison of key signaling networks between subgroups. CellChat analysis predicted that multiple key networks, including the EGF **(F)**, MIF** (G)**, CD40 **(H)**, and CypA **(I)** pathways, were more prominently associated with the signature-high group.

Building on these findings, we next examined the predicted intercellular communication patterns of signature-high malignant cells within the TME. Signature-based scoring separated malignant cells into signature-high and signature-low populations (Figure 6A), and their intercellular communication networks were subsequently inferred using CellChat. The TME displayed extensive intercellular signaling, with both the quantity and overall strength of inferred interactions being elevated across malignant, stromal, and immune cell populations (Figure 6B-C). Analysis of ligand-receptor pairs suggested broader predicted crosstalk between signature-high malignant cells and immune or stromal populations than that observed in signature-low malignant cells (Figure 6D-E).

The signature-high group showed higher predicted outgoing signaling than the signature-low group (Figure 6F). This pattern was observed across multiple pathways, with signature-high malignant cells predicted to act as the predominant sender (Figure 6D, F-G). EGF signaling was prominently associated with the signature-high group (Figure 6F). Meanwhile, TGFB, MIF, CD40, and CypA signaling also showed stronger predicted activity in signature-high malignant cells (Figure 6D, G-I).

Together, these inferred signaling differences suggest a potentially pro-tumorigenic communication profile associated with the signature-high state, which warrants further experimental validation.

### The ac4C-Related Signature Predicts Therapeutic Sensitivities

**Figure 7:**
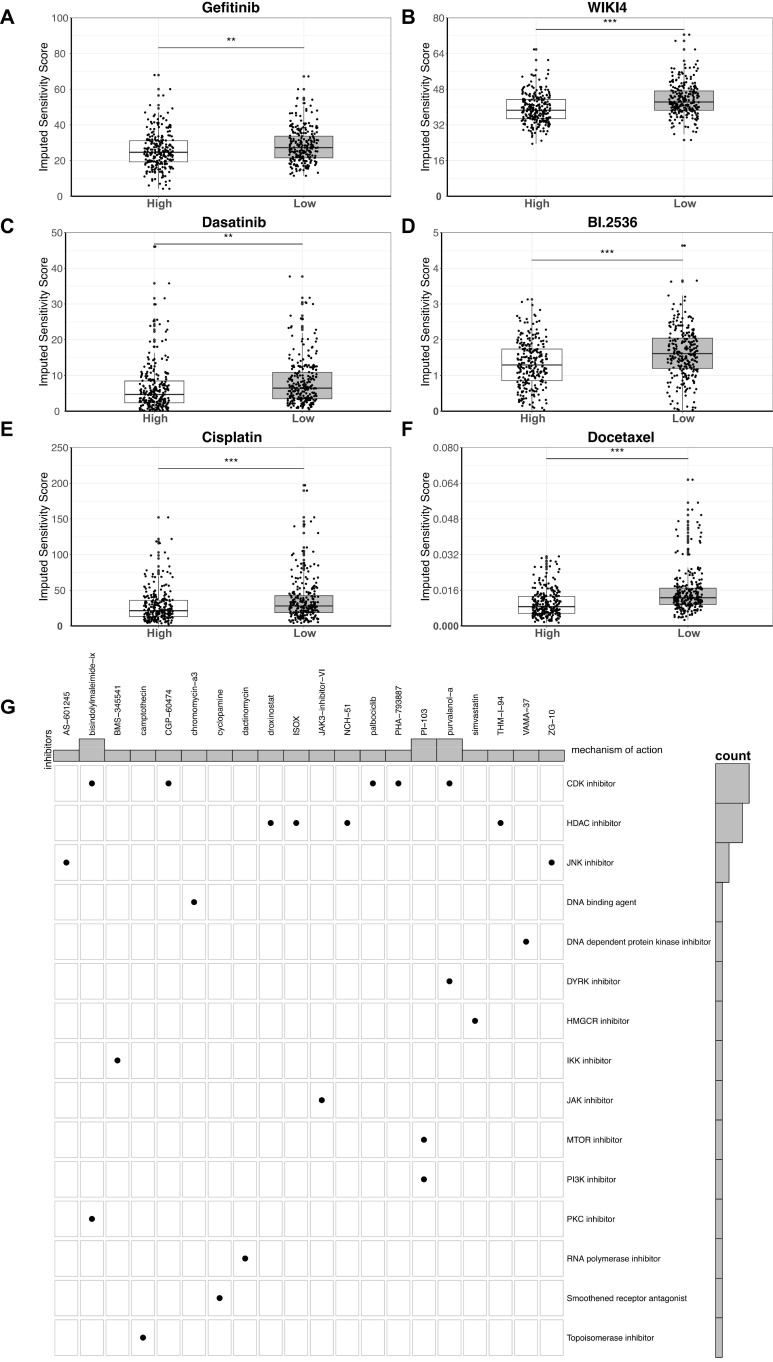
***In silico* prediction of drug sensitivity in the high-risk subgroup. (A-F)** Boxplots showing the Imputed Sensitivity Scores of six therapeutic agents in the high-risk group, with the low-risk group shown for comparison. A lower score indicates greater sensitivity. The agents shown are Gefitinib **(A)**, WIKI4 **(B)**, Dasatinib** (C)**, BI.2536** (D)**, Cisplatin **(E)**, and Docetaxel **(F)**. Statistical significance is denoted by asterisks (**P < 0.01 and ***P < 0.001). **(G)** An overview of a drug screen identifying classes of compounds predicted to be associated with the high-risk subgroup. The plot shows specific inhibitors (columns) and their corresponding mechanisms of action (rows). The bar chart on the right indicates the number of drugs identified within each mechanistic class.

To explore potential treatment-related hypotheses, we performed *in silico* drug sensitivity prediction in the high-risk subgroup. Pharmacogenomic modeling suggested potentially increased sensitivity of the high-risk subgroup to several therapeutic agents, including gefitinib, dasatinib, cisplatin, and docetaxel (Figure 7A-F).

Based on these results, we next performed a computational drug screening analysis to identify additional compound classes potentially associated with the high-risk subgroup. The analysis identified multiple classes of inhibitors with predicted efficacies, among which, specific inhibitors of CDK (Cyclin-dependent kinases) (such as Palbociclib) and HDAC (Histone deacetylases) were most enriched categories (Figure 7G). These findings nominate candidate treatment hypotheses for the high-risk subgroup, although further experimental and clinical validation is required.

### PLK1 Knockdown Suppresses LUAD Cell Proliferation and Migration

**Figure 8:**
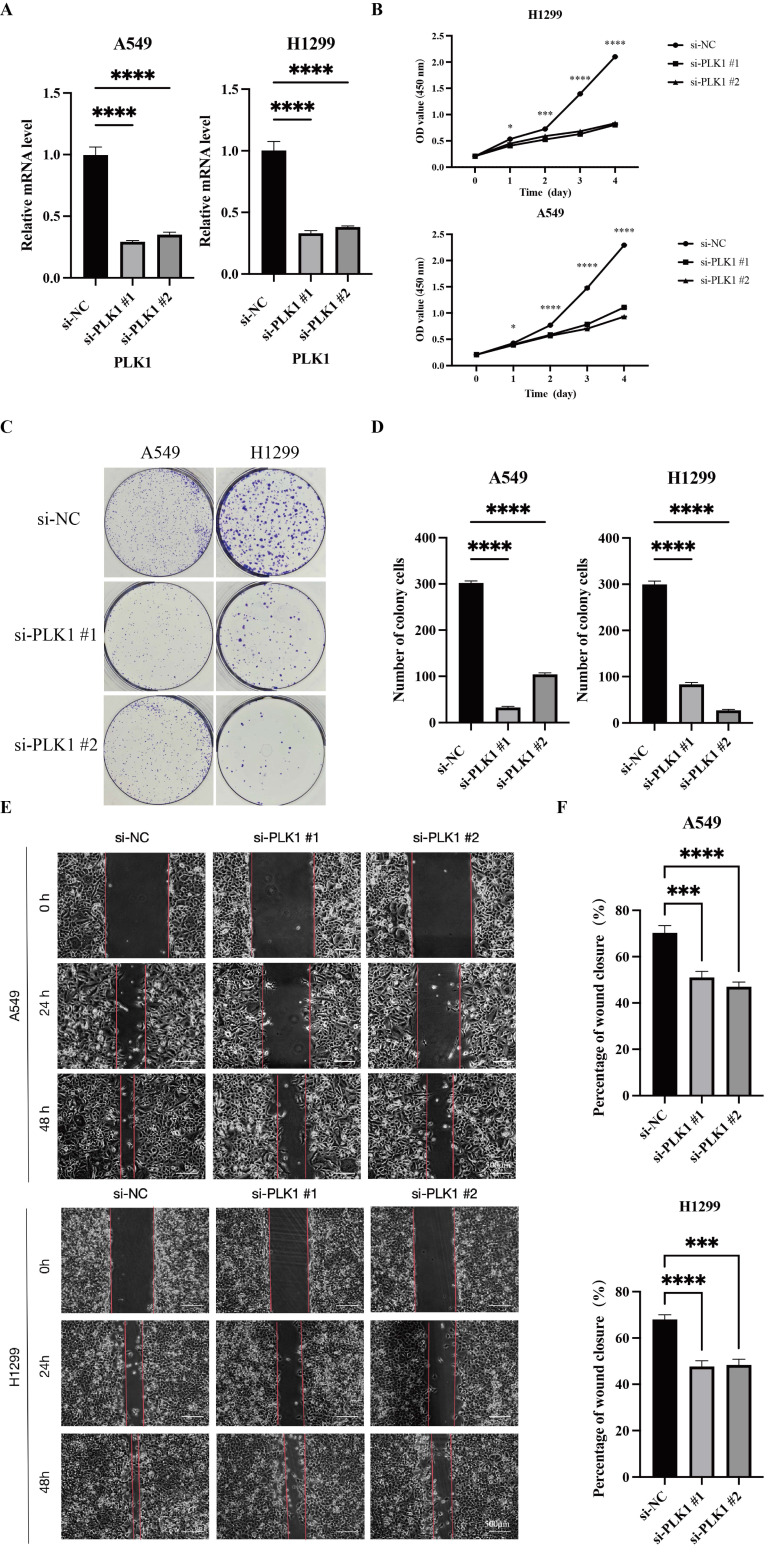
** PLK1 is required for LUAD cell proliferation and migration. (A)** RT-qPCR validation of PLK1 knockdown by two independent siRNAs (si-PLK1 #1/#2) in A549 and H1299 cells. **(B)** CCK-8 assays assessing cell proliferation after PLK1 silencing in A549 and H1299 cells. **(C, D)** Colony formation assays **(C)** and quantification of colony numbers **(D)** following PLK1 knockdown. **(E, F)** Wound-healing assays in A549 **(E)** and H1299 **(F)** cells, with representative images at the indicated time points and quantification of wound closure. Data are presented as mean ± SD. ns, not significant; *P < 0.05, **P < 0.01, ***P < 0.001, ****P < 0.0001.

Among the six signature genes, PLK1 was prioritized for validation because it showed the strongest differential expression and has established relevance to cell-cycle regulation and therapeutic targeting. RT-qPCR confirmed the efficiency of PLK1 knockdown in A549 and H1299 cells (Figure 8A). PLK1 knockdown reduced proliferative capacity, as shown by the CCK-8 assay (Figure 8B) and decreased clonogenic growth in colony formation assays (Figure 8C-D). Moreover, wound-healing assays showed markedly attenuated migratory capacity upon PLK1 knockdown in both cell lines (Figure 8E, F). Collectively, these results support PLK1 as a functional contributor to the aggressive signature-high phenotype and a candidate therapeutic target in LUAD.

## Discussion

N4 acetylcytidine (ac4C) represents a form of RNA acetylation that has recently gained attention within epitranscriptomics. Reported functions of ac4C include modulation of mRNA stability and translation, and dysregulation of this mark has been implicated in the pathogenesis of several malignancies^9^. Accumulating evidence indicates that ac4C modification participates in diverse biological programs and contributes to cancer initiation and progression, influencing processes such as cell growth and metastasis, metabolic reprogramming, and treatment resistance^14^. The ac4C modification has been increasingly implicated in cancer initiation and therapeutic response, suggesting potential translational relevance. Nevertheless, compared with better characterized RNA marks such as m6A, the functional roles and regulatory architecture of ac4C in LUAD remain insufficiently defined, particularly within the tumor microenvironment and in the setting of marked intratumoral heterogeneity.

In this study, our integrative analysis identified an ac4C-related transcriptomic signature associated with LUAD progression. The novelty of this study lies in establishing a machine-learning-derived ac4C signature for prognostic stratification and linking it to an aggressive malignant program at single-cell resolution. Furthermore, we found that malignant cells with high ac4C-related signature scores were associated with enhanced pro-tumorigenic signaling within the TME through enhanced EGF and TGFB signaling, and then translated these findings by identifying a clinically actionable vulnerability to CDK and PLK1 inhibitors in the high-risk subgroup.

We started with an unbiased genome-wide analysis using WGCNA and identified two modules (MEblue and MEgreen) that were significantly associated with adverse clinical outcomes in lung adenocarcinoma. According to functional enrichment analysis, the two modules participated in divergent biological processes: MEblue was mainly enriched in cell cycle and proliferation, while MEgreen was mainly enriched in RNA processing and cell adhesion. This finding indicates that there may exist multiple and distinct biological processes involved in the aggressive phenotype of LUAD. To convert these module-level findings into a clinically applicable tool, we employed a multi-algorithm machine learning strategy. By taking the consensus of LASSO, Random Forest, and SVM-RFE, we extracted a robust six-gene ac4C-related signature. Here, “ac4C-related” indicates that the model was developed from ac4C-associated transcriptomic programs and was supported by its correlation with NAT10 expression, rather than direct evidence of ac4C modification of each signature gene. Given the enrichment of cell-cycle and oncogenic pathways, this signature may reflect both ac4C-related regulatory features and broader malignant proliferative programs associated with LUAD progression.

After identifying the prognostic signature, we characterized the biological profile of the high-risk subgroup it defines. Gene Set Enrichment Analysis showed significant enrichment of hallmark oncogenic pathways (E2F Targets, MYC Targets, and mTORC1 signaling) and of metabolic programs (Glycolysis and Oxidative Phosphorylation), indicating pronounced metabolic reprogramming. We then interrogated the somatic mutation landscape and observed a distinct profile in the high-risk subgroup, with higher frequencies of TP53, CSMD3, PCLO, and SPTA1 mutations, whereas DZIP1 mutations were enriched in the low-risk group—findings consistent with large-scale LUAD genomics^45^. Notably, CSMD3 mutation status has been associated with tumor proliferation and the timing of PCLO mutations with prognosis; In addition, the high-risk subgroup showed a higher burden of concurrent mutations, which is often associated with aggressive behavior and genomic instability. Together, these findings suggest that the ac4C-related high-risk state is accompanied by a distinct somatic mutation profile, providing a genomic explanation for the unfavorable clinical outcome.

To elucidate the cellular basis of the bulk level phenotypes observed in the LUAD tumor microenvironment, we performed single-cell transcriptomic analyses. Bulk RNA-seq analyses can provide rich information about the heterogeneity within a malignant cell population, but these studies remain limited because the malignant cell population itself harbors substantial heterogeneity^38^. In contrast, single-cell RNA-seq analyses can dissect heterogeneity within the malignant cell population itself, which represents a major advance of our study. By applying our signature score to individual cells, we discovered a specific subpopulation of malignant cells (clusters 2, 4, and 6) characterized by high ac4C-related signature scores. Pseudotime trajectory analysis suggested that signature-high malignant cells were enriched toward a terminal region of an inferred trajectory, potentially reflecting a more advanced malignant state. Because PLK1 and CDCA4 are cell-cycle-associated genes, the signature-high state may partly reflect a proliferative malignant program rather than ac4C-specific regulation alone. Consistent with this interpretation, the enriched programs included ribosome biogenesis, a hallmark of enhanced protein synthesis required for rapid cellular proliferation^46^. This important functional insight was further supported by the observation that key cell cycle regulators from our signature (PLK1, CDCA4) were highly expressed along this developmental trajectory.

In addition to their intrinsic characteristics, cancer cells also modulate their microenvironment to support their survival and proliferation. Our cell-cell communication analysis suggested that signature-high malignant cells may serve as important predicted signaling sources within the TME. Signature-high malignant cells showed globally enhanced predicted signaling output in pathways involved in LUAD progression. EGF signaling was predicted to be prominently associated with signature-high malignant cells and their interactions with fibroblasts and endothelial cells^47^. Interestingly, the strong activation of the immunosuppressive TGFB pathway in the same group of cells provides a mechanism by which this tumor may escape from immune surveillance^48^. The activation of other pro-tumorigenic pathways like MIF and CypA support the conclusion that the signature-high phenotype was associated with a broadly pro-tumorigenic communication landscape^49^, ^50^.

Finally, we investigated the clinical relevance of our ac4C signature to guide personalized therapy. We found that the high-risk subgroup, although with highly aggressive biology, has certain therapeutic weaknesses. This group was significantly sensitive to known agents, such as EGFR inhibitor Gefitinib and conventional chemotherapy drugs like Cisplatin^51^. This is consistent with the biology of highly proliferative tumors which are more sensitive to DNA damaging agents and cell cycle inhibitors. More importantly, our comprehensive drug screen discovered novel therapeutic approaches for this clinically challenging subgroup. The predicted sensitivity to CDK inhibitors (e.g. Palbociclib) and HDAC inhibitors provide solid rationale to test these drugs in patients stratified by our ac4C signature^52^, ^53^. Our drug response inference suggested that the high-risk subgroup may exhibit increased sensitivity to BI-2536, a Polo-like kinase inhibitor^54^, ^55^. This observation is consistent with the inclusion of PLK1 in our signature, as PLK1 is a key cell-cycle regulator and a known target of BI-2536^42^. This finding provides a computationally supported therapeutic hypothesis. Since CDK and PLK1 inhibitors target the cell cycle machinery that are highly activated in this subgroup, this suggests a potential therapeutic strategy that warrants further validation.

This study has several limitations. First, the ac4C-related signature was developed and evaluated using retrospective public cohorts, and its prognostic value and utility for treatment stratification require validation in independent prospective clinical datasets. Second, the pseudotime, cell-cell communication, and drug sensitivity analyses are computationally inferred and should be considered hypothesis-generating pending experimental validation. In addition, the inferred pseudotime trajectory may be affected by root-state selection, and sensitivity analyses using alternative trajectory settings are needed to further confirm its robustness. Third, this study identifies an ac4C-related transcriptomic signature rather than directly demonstrating ac4C modification of the six signature genes. Additional analyses adjusted for proliferation or cell-cycle activity will be needed to further separate ac4C-related regulatory signals from general proliferative programs. Although NAT10 expression was positively correlated with the risk score, NAT10 perturbation experiments or acRIP-based assays are needed to clarify the mechanistic basis of this association. Because the functional validation was limited to PLK1 knockdown in two LUAD cell lines, further rescue experiments, pharmacological inhibition assays, *in vivo* studies, and clinical validation are needed to strengthen the mechanistic and translational significance of our findings.

Overall, our integrative transcriptomic analysis identified an ac4C-related signature in LUAD that stratified patients and was associated with aggressive malignant states and predicted communication patterns within the TME, highlighting potential therapeutic vulnerabilities. Notably, PLK1 was functionally validated *in vitro*, supporting the biological and translational relevance of this signature.

## Supplementary Material

Supplementary figures and tables.

## Data Availability

The public datasets were obtained from TCGA via Xena platform (https://xena.ucsc.edu/), DepMap portal (https://depmap.org/portal/), Code Ocean capsule database (10.24433/CO.0121060.v1).

## Abbreviations

LUAD: lung adenocarcinoma
ac4C: N4-acetylcytidine
NAT10: N-acetyltransferase 10
TME: tumor microenvironment
RNA-seq: RNA sequencing
scRNA-seq: single-cell RNA sequencing
TCGA: The Cancer Genome Atlas
GEO: Gene Expression Omnibus
TPM: transcripts per million
WGCNA: weighted gene co-expression network analysis
DEGs: differentially expressed genes
log2FC: log2 fold-change
GO: Gene Ontology
KEGG: Kyoto Encyclopedia of Genes and Genomes
GSEA: Gene Set Enrichment Analysis
CNV: copy number variation
GDSC: Genomics of Drug Sensitivity in Cancer
IC50: half maximal inhibitory concentration
OS: overall survival
HR: hazard ratio
UMAP: Uniform Manifold Approximation and Projection
LASSO: least absolute shrinkage and selection operator
RF: random forest
SVM-RFE: support vector machine-recursive feature elimination
EGF: epidermal growth factor
EGFR: epidermal growth factor receptor
TGF-β: transforming growth factor-β
CDK: cyclin-dependent kinase
PLK1: polo-like kinase 1
HDAC: histone deacetylase
