# Supplementary material for: Integrative bulk and single-cell transcriptomic analysis identify an ac4C-related signature in lung adenocarcinoma
Source: J Cancer. 2026 Jul 13;17(7):1331–45. doi: 10.7150/jca.135531 (PMC13410428; doi:10.7150/jca.135531)
Supplement: Supplementary file 1 — Supplementary figures and tables. [file jcav17p1331s1.pdf]

Supplementary Figure S1

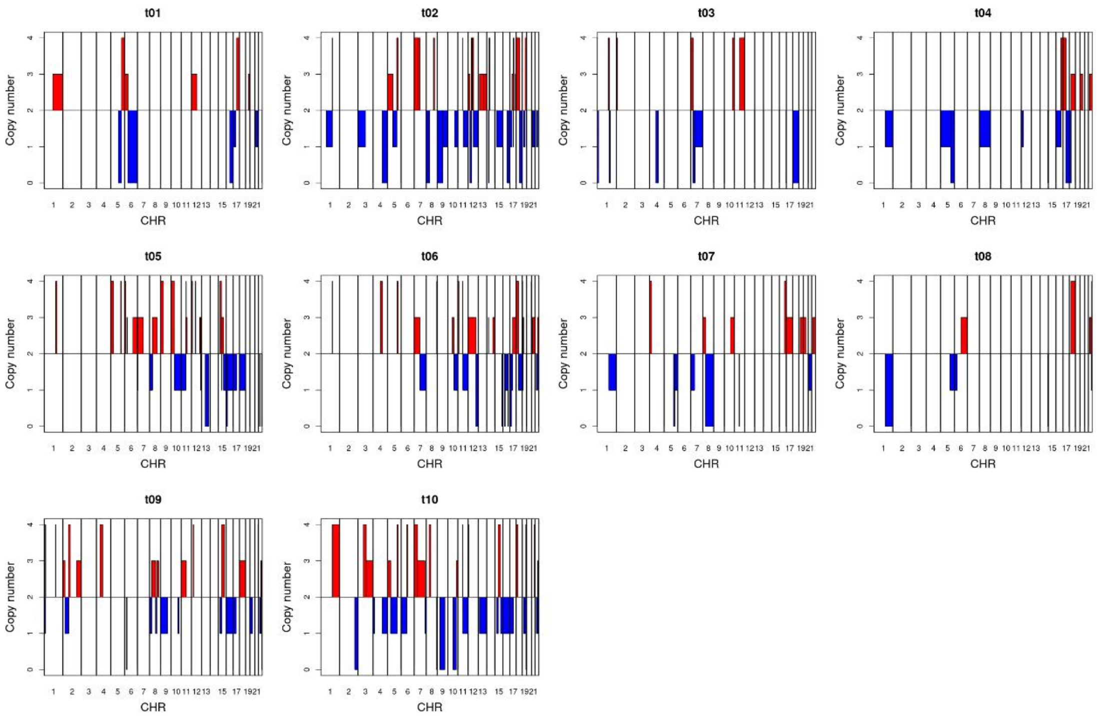

Figure S1. Genome-wide copy-number profiles of tumor cells from all 10 LUAD samples. Each panel (t01–t10) displays copy-number variation of genes along the chromosomes (x-axis, CHR 1–21), with the y-axis indicating inferred copy-number states. Red bars represent copy-number gains and blue bars represent copy-number losses.

Supplementary Figure S2

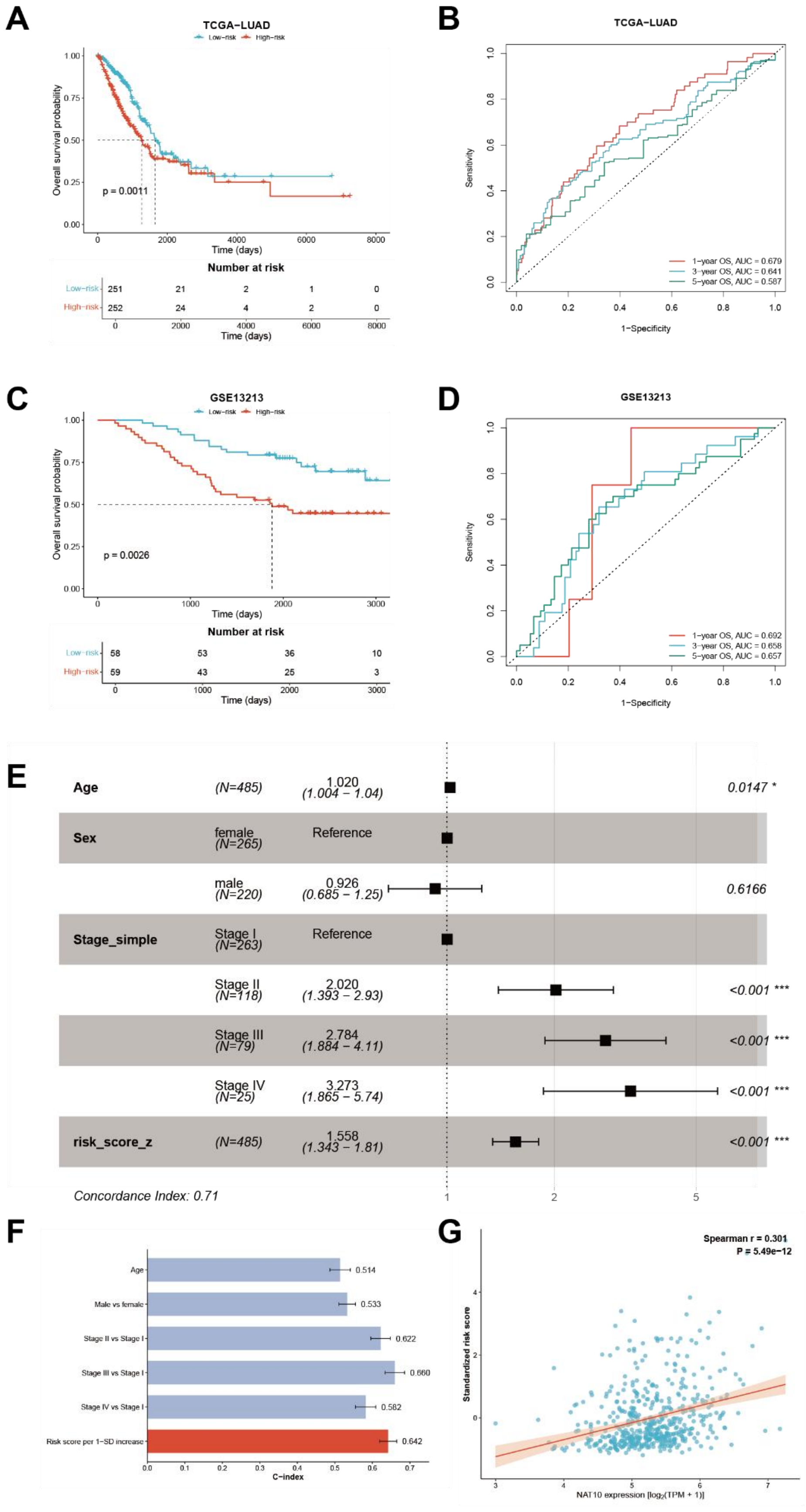

Figure S2. Additional validation and prognostic evaluation of the six-gene ac4C-related signature in LUAD. (A, B) Kaplan–Meier survival curve and time-dependent ROC analysis of the six-gene signature in the TCGA-LUAD cohort. **(C, D)** Kaplan–Meier survival curve and time-dependent ROC analysis of the signature in the independent GSE13213 cohort. **(E)** Multivariate Cox regression analysis of overall survival in the TCGA-LUAD cohort. **(F)** Comparison of C-index values from six univariate Cox models in the TCGA-LUAD cohort. **(G)** Spearman correlation analysis between NAT10 expression and the standardized risk score in TCGA-LUAD.

Supplementary Table S1. Prognostic validation of the six-gene signature

Cohort	N	HR per 1-SD increase in risk score	95% CI	*P* value	C-index	1-year AUC	3-year AUC	5-year AUC
GSE13213	117	1.387	1.068–1.801	0.0142	0.623	0.692	0.658	0.657
TCGA-LUAD internal validation	503	1.593	1.393–1.821	1.04E-11	0.629	0.679	0.641	0.587

HR, hazard ratio; **CI**, confidence interval; **AUC**, area under the curve.

Supplementary Table S2. Multivariate Cox regression analysis of overall survival in the TCGA-LUAD cohort

**Variable**	**HR**	**95% CI**	***P value***
Age	1.020	1.004–1.036	0.0147
Sex: male vs female	0.926	0.685–1.252	0.617
Stage II vs Stage I	2.020	1.393–2.930	0.000209
Stage III vs Stage I	2.784	1.884–4.114	2.74×10^{-7}
Stage IV vs Stage I	3.273	1.865–5.743	3.58×10^{-5}
Risk score per 1-SD increase	1.558	1.343–1.806	4.60×10^{-9}

HR, hazard ratio; **CI**, confidence interval. Female sex and Stage I were used as the reference categories. The analysis included 485 patients with complete clinicopathological information.

Supplementary Table S3. Summary of the six genes in the ac4C-related prognostic signature

Gene	Chromosomal location	Established biological function	Reported cancer-related role	Representative references
**CDCA4**	14q32.33	E2F-responsive nuclear factor involved in E2F-dependent transcriptional regulation and cell proliferation.	Reported to promote LUAD proliferation through interaction with IGF2BP1 and activation of the PI3K/AKT pathway; related to cell-cycle-dependent tumor growth programs.	[1, 2]
**GCLC**	6p12.1	Catalytic subunit of glutamate-cysteine ligase, the rate-limiting enzyme for glutathione biosynthesis and cellular redox homeostasis.	Supports antioxidant and redox adaptation in cancer cells; glutathione-related pathways contribute to tumor progression, therapy resistance, and cisplatin resistance in LUAD.	[3, 4]
**HMGA1**	6p21.31	AT-hook chromatin architectural protein involved in chromatin organization and transcriptional regulation.	Implicated in oncogenic transcriptional programs, chromatin organization, Wnt-associated signaling, lipid metabolic reprogramming, and tumor progression.	[5-7]
**PLK1**	16p12.2	Mitotic serine/threonine kinase involved in G2/M progression, spindle formation, chromosome segregation, and cytokinesis.	A well-established cancer-associated kinase and therapeutic target; reported to promote Kras/Tp53-mutant LUAD development through RET/MAPK signaling.	[8, 9]
**SLC7A5**	16q24.2	L-type amino acid transporter 1 mediating uptake of large neutral amino acids and supporting nutrient-dependent growth signaling.	Promotes cancer cell growth through amino acid transport and mTORC1-related metabolic signaling; reported as a LUAD-specific prognostic biomarker associated with an immunosuppressive tumor microenvironment.	[10, 11]
**TNNT1**	19q13.42	Slow skeletal troponin T isoform and tropomyosin-binding component of the troponin complex involved in thin-filament regulation.	Reported to promote migration, invasion, and epithelial-mesenchymal transition in lung cancer cells, potentially through Wnt/β-catenin signaling.	[12, 13]

Note. Chromosomal locations were annotated according to public gene annotation resources. Biological functions and cancer-related roles were summarized from representative published studies.

Abbreviations: LUAD, lung adenocarcinoma; EMT, epithelial-mesenchymal transition; GSH, glutathione; LAT1, L-type amino acid transporter 1; mTORC1, mechanistic target of rapamycin complex 1.

References

1. Hayashi R, Goto Y, Ikeda R, Yokoyama KK, Yoshida K. CDCA4 is an E2F transcription factor family-induced nuclear factor that regulates E2F-dependent transcriptional activation and cell proliferation. J Biol Chem. 2006; 281: 35633-48.
2. Feng S, Cao H, Sui Y, Shen Z, Wu J, Ma R, et al. CDCA4 interacts with IGF2BP1 to regulate lung adenocarcinoma proliferation via the PI3K/AKT pathway. Thorac Cancer. 2023; 14: 724-35.
3. Harris IS, Treloar AE, Inoue S, Sasaki M, Gorrini C, Lee KC, et al. Glutathione and thioredoxin antioxidant pathways synergize to drive cancer initiation and progression. Cancer Cell. 2015; 27: 211-22.
4. Traverso N, Ricciarelli R, Nitti M, Marengo B, Furfaro AL, Pronzato MA, et al. Role of glutathione in cancer progression and chemoresistance. Oxid Med Cell Longev. 2013; 2013: 972913.
5. Xian L, Georgess D, Huso T, Cope L, Belton A, Chang YT, et al. HMGA1 amplifies Wnt signalling and expands the intestinal stem cell compartment and Paneth cell niche. Nat Commun. 2017; 8: 15008.
6. Luo LZ, Kim JH, Herrera I, Wu S, Wu X, Park SS, et al. HMGA1 acts as an epigenetic gatekeeper of ASCL2 and Wnt signaling during colon tumorigenesis. J Clin Invest. 2025; 135.

7. Zhao Y, Liu MJ, Zhang L, Yang Q, Sun QH, Guo JR, et al. High mobility group A1 (HMGA1) promotes the tumorigenesis of colorectal cancer by increasing lipid synthesis. Nat Commun. 2024; 15: 9909.
8. Strebhardt K. Multifaceted polo-like kinases: drug targets and antitargets for cancer therapy. Nat Rev Drug Discov. 2010; 9: 643-60.
9. Kong Y, Allison DB, Zhang Q, He D, Li Y, Mao F, et al. The kinase PLK1 promotes the development of Kras/Tp53-mutant lung adenocarcinoma through transcriptional activation of the receptor RET. Sci Signal. 2022; 15: eabj4009.
10. Kanai Y. Amino acid transporter LAT1 (SLC7A5) as a molecular target for cancer diagnosis and therapeutics. Pharmacol Ther. 2022; 230: 107964.
11. Wang Q, Holst J. L-type amino acid transport and cancer: targeting the mTORC1 pathway to inhibit neoplasia. Am J Cancer Res. 2015; 5: 1281-94.
12. Wei B, Jin JP. TNNT1, TNNT2, and TNNT3: Isoform genes, regulation, and structure-function relationships. Gene. 2016; 582: 1-13.
13. Ge X, Du G, Zhou Q, Yan B, Yue G. TNNT1 accelerates migration, invasion and EMT progression in lung cancer cells. Thorac Cancer. 2024; 15: 1749-56.
